# Understanding maternal postnatal blood pressure changes following hypertensive disorders in pregnancy: protocol for a prospective cohort study

**DOI:** 10.1136/bmjopen-2021-060087

**Published:** 2022-04-01

**Authors:** Elaine Sheehan, Chao Wang, Matthew Cauldwell, Debra Bick, Basky Thilaganathan

**Affiliations:** 1 Molecular and Clinical Sciences Research Institute, St George's University of London Molecular and Clinical Sciences Research Institute, London, UK; 2 Maternal Medicine, St George's University Hospitals NHS Foundation Trust, London, UK; 3 Faculty of Health, Social Care and Education, Kingston University and St. George's, University of London, London, UK; 4 Warwick Clinical Trials Unit, Warwick Medical School, University of Warwick, Coventry, West Midlands, UK; 5 Fetal Medicine, St George's University of London Molecular and Clinical Sciences Research Institute, London, UK

**Keywords:** hypertension, maternal medicine, obstetrics

## Abstract

**Introduction:**

Hypertensive disorders occur in approximately 10% of women during pregnancy. There is robust population-based data to show that women who have hypertension in pregnancy are much more likely to develop cardiovascular disease (CVD) in the postpartum period. Women with a hypertensive disorder of pregnancy (HDP) are twice more at risk of heart disease and stroke, and four times more likely to develop hypertension after birth. Two out of three women who had HDP will die from CVD. Recent evidence suggests that young women with HDP develop signs of CVD in the immediate postpartum period, rather than several decades later as previously presumed. If confirmed, this concerning finding presents healthcare practitioners with an opportunity to influence women’s cardiovascular health by advising on lifestyle choices and considering therapeutic interventions to prevent the development of CVD.

**Methods and analysis:**

This prospective cohort study design will ask approximately 300 participants to complete 3 days of home blood pressure monitoring every fortnight for 12 weeks postpartum and will culminate with a 24-hour episode of ambulatory blood pressure monitoring at 12 weeks postpartum. Women and healthcare professionals will complete questionnaires surrounding postpartum care for women who had HDP and knowledge of CVD risk. In addition, the relationship between hypertension and factors likely to influence outcomes such as severity of HDP, maternal age, body mass index and ethnicity will be analysed using logistic regression. Blood pressure and data from questionnaires will be analysed using descriptive statistics, with temporal stratification.

**Ethics and dissemination:**

Research ethics approval was obtained from London-West London & GTAC Research Ethics Committee. Research outputs will be published and disseminated through midwifery, obstetric or general practitioner targeted academic journals. The patient and public involvement group will disseminate findings to women who have experienced HDP among their peer groups.

**Trial registration number:**

NCT05137808.

**Strengths and limitations of this study**

- This is the first study to our knowledge exploring the recovery of blood pressure longitudinally in the immediate postpartum period after gestational hypertension.
- Home blood pressure monitoring will be compared against the gold standard; ambulatory blood pressure monitoring.
- Self-monitoring and reporting of blood pressure measurements by research participants may introduce the challenge of missing data.

## Introduction

Hypertensive disorders of pregnancy (HDP) affect approximately 10% of pregnant women.^1^ The most serious of these disorders is pre-eclampsia which affects 3%–5% of pregnant women worldwide. Aside from chronic or pre-existing hypertension which predates pregnancy, gestational hypertension and pre-eclampsia can occur at any point in pregnancy and early postpartum period from 20 weeks gestation.^1^ Women who experience HDP are at increased risk of cardiovascular disease (CVD), hypertension, diabetes, renal disease and stroke in their future life.^1 2^ Hypertension is the leading risk factor for CVD in the UK^3^ and it can be identified as early as the immediate postpartum period.^4–6^ Recent evidence suggests that young women are developing signs of CVD immediately in the postpartum period after HDP, rather than several decades later than previously presumed.^6 7^

CVD is largely preventable with simple lifestyle modifications and treatment.^8^ If postpartum women with a history of HDP engaged in healthy lifestyle activities (moderate exercise, reduced alcohol consumption, stopped smoking, healthier diets, reduce time watching television and maintained a healthy body mass index <25), they could reduce their risk of CVD by up to 92%.^9^

There is currently no standard monitoring for CVD development postnatally for women who had HDP, unlike their counterparts—women who develop gestational diabetes (GDM). Women with GDM are at increased risk of type 2 diabetes in later life. In the postpartum period, general practitioners (GPs) assess fasting blood glucose and/or haemoglobin A1c to identify the development of type 2 diabetes for women who had GDM and they will receive this annually.^10^ Women who have HDP currently do not receive this level of monitoring for the development of CVD. This study seeks to address this disparity by assessing the prevalence of postnatal hypertension, which may highlight the need for monitoring of CVD development.

The gold standard for diagnosing hypertension is 24-hour ambulatory blood pressure monitoring (ABPM).^11^ However, home blood pressure monitoring (HBPM) is becoming more widely used in maternity care, particularly in the antenatal period since the COVID-19 pandemic^12^ and more recently, self-management of BP in the postpartum period has been considered.^13^ To add to this expanding evidence-base, this study will assess the accuracy of HBPM versus ABPM and if there is a role for HBPM in the postnatal period for diagnosing hypertension.

This study will highlight the proportion of women with persistent hypertension from week 2 to 12 postpartum and whether these women share common characteristics such as raised body mass index and/or maternal age. This is valuable information for clinicians so they know which women are at higher of risk of uncontrolled hypertension and require closer blood pressure (BP) surveillance. The current national guidance for monitoring BP in the postpartum period is minimal and this research will therefore ascertain if the national guidance for monitoring hypertension^14^ in the postpartum period is sufficient. It will highlight if women who had HDP need further monitoring of hypertension between week 2 and week 8 postpartum and therefore highlight the important timelines for monitoring BP and the need for therapeutic intervention to control BP.

This study will ascertain if the HCPs involved in the care of women who may at be at increased risk of CVD due to developing HDP are educated about the risks and approaches to monitor for CVD. Roth *et al* undertook a systematic review assessing knowledge gaps of women and HCPs around CVD risk and HDP.^15^ The authors concluded that there was a low level of knowledge among women and HCPs concerning CVD risk and HDP. If HCPs and women are not educated about the risks, the path to significant heart disease is expedited. The postnatal period presents an optimum time for women to be receptive to change and lifestyle modifications,^16^ with opportunities for HCPs to influence positive change.

The main objective of this prospective cohort study is to observe the recovery (or lack thereof) of maternal hypertension from week 2 to week 12 after a pregnancy affected by new onset of hypertension and identify the prevalence of persistent postpartum hypertension in this cohort of women. A secondary objective will be to assess the accuracy of HBPM in comparison with ABPM to inform if HBPM can be used as an alternative method for diagnosing hypertension in the postpartum period. Other outcomes of interest will include women’s self-reporting of their 6-week to 8-week GP postnatal review, their perceived risk of developing CVD associated with HDP, their emotional well-being and their views on HBPM and ABPM. These objectives will be assessed via online questionnaires distributed at 8 and 12 weeks post partum, respectively. In addition, healthcare professionals involved in the postnatal care of women with HDP will receive an online questionnaire assessing their knowledge on HDP and associated CVD risk.

## Methods and analysis

### Recruitment and sampling

Participants will be recruited across three NHS maternity sites in South England. Potential participants will be approached during the antenatal and postnatal period before discharge home if they have been identified as having new onset of hypertension in pregnancy by way of convenience sampling. Recruitment will commence in April 2022 until March 2023 and all data collection will cease in June 2023 allowing 12 weeks of BP monitoring from when final participants were recruited in March 2023.

### Sample size

To obtain an accurate estimate of persistent hypertension rate after birth, a sample size of 263 women with a history of HDP are required. The target sample size is 300 women to compensate for study withdrawal and incomplete BP data. This is based on the anticipated rate of persistent postpartum hypertension of approximately 20% and the corresponding width of 95% CI to be 10% as desired level of precision, with 80% probability of achieving such precision. Considerations were also given to the sample size required to assess agreement between HBPM and ABPM.

### Inclusion and exclusion criteria

Eligible research participants will require a diagnosis of new-onset hypertension in pregnancy after 20 weeks gestation and up to 72 hours postpartum. Research participants in whom English is not their primary spoken language must demonstrate evidence of the ability to communicate any abnormal BP readings with their healthcare provider.

### Patient and public involvement

The patient and public involvement (PPI) steering group includes four women from different ethnic groups including black Caribbean, Asian and white British and European, with a lived experience of HDP who were all involved in discussions related to research priorities and the development of the proposed research study to address priority questions. The PPI group experienced a range of BP disorders during pregnancy including pre-existing hypertension, pre-eclampsia and gestational/postnatal hypertension. This diverse mix of service user backgrounds and experiences ensured that the research team can gain insight into how women prioritise their physical and mental health and well-being after birth, and extent to which this may be influenced by their ethnic and cultural backgrounds.

The PPI group have reviewed the research protocol and all research materials (participant information leaflet, consent forms, questionnaires, posters and BP monitoring instructions, study diary), which were revised to reflect their preferences including how information was structured and ensuring language used is suitable for a lay audience. The PPI group have informed the researcher on all stages of the research project since inception, with financial remuneration for their input and time made in line with the National Institute of Health Research guidance.

### Data collection

#### Home blood pressure monitoring

**Figure 1:**
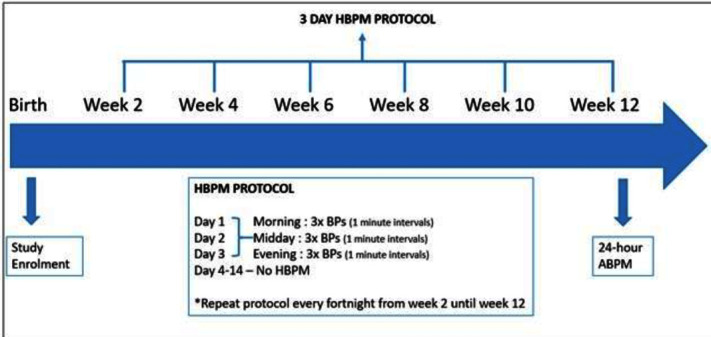
Overview of participant blood pressure (BP) monitoring protocol. ABPM, ambulatory blood pressure monitoring; HBPM, home blood pressure monitoring.

Research participants will undergo HBPM every fortnight from week 2 post birth until week 12 as per a HBPM protocol based on that used by Alemida *et al*. Women will take three BP readings in the morning, early afternoon and in the evening, a total of nine BP readings per day, for three consecutive days.^17^ At week 12, women will undergo 24-hour ABPM (figure 1).

HBPM will be undertaken using Microlife Watch BP Home as they have been validated for pregnancy and pre-eclampsia.^18^ Participants will record their BP measurements manually in a diary provided by the research team. Participants will receive guidance on what to do if they discover BP outside the normal range. Local research midwives at each study site will upload the BP measurements directly from the BP monitor into an electronic file on return of the BP diaries and monitor at 12 weeks postpartum.

#### Ambulatory blood pressure monitoring

Ambulatory BP monitoring will be undertaken using Mobil-O-Graph as this monitor is endorsed by the British and Irish Hypertension Society and has been validated against the European Society of Hypertension criteria.^19 20^ The study site research midwives will upload the 24-hour BP analysis to a password-protected excel file.

#### Questionnaires

**Figure 2:**
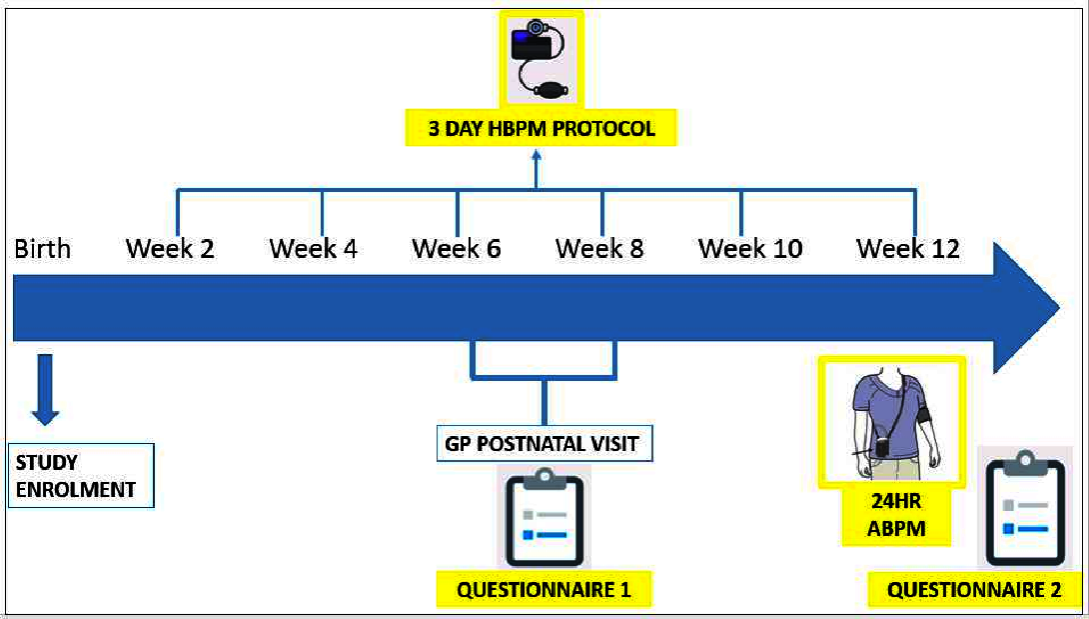
Timeline of events for research participant. ABPM, ambulatory blood pressure monitoring; GP, general practitioner; HBPM, home blood pressure monitoring.

1. Questionnaire 1 will be distributed online at 8 weeks postpartum to assess if participants had a postnatal GP review and self-report on what occurred at this appointment if applicable. Part 2 of the questionnaire will assess participants’ risk perception of CVD development and emotional well-being following a pregnancy with HDP.
2. Questionnaire 2 will be distributed at 12-week post partum assessing participants’ views on their experience of HBPM and ABPM (figure 2).
3. A questionnaire aimed at clinicians involved in the care of postpartum women with a HDP will be distributed to midwives, obstetricians and GPs assessing their knowledge on HDP and associated risk of CVD. In addition, a focus group with GPs will provide insight to the postnatal GP check from a clinician’s perspective.

### Data analysis

#### Objective 1: to identify which BP will be used to calculate the rate of persistent hypertension at 12 weeks postpartum

The first and second BP readings will be compared with the third reading to assess if they vary by >10/5 mm Hg. The mean or median of all BPs will be used if no significance is found or the third BP reading will be used for interpretation if there is a significant difference (>10/5 mm Hg) between BPs. The BP taken at three time periods (morning, midday and evening) will also be analysed to assess if findings vary significantly by >10/5 mm Hg. If no significance is found, the mean or median BP readings for the day will be calculated and used for further analysis. If significant difference found, each time period will be compared with daytime ABPM to see which time point mostly correlates with daytime ABPM. This will be analysed using one sample t-test of the difference and intraclass correlation co-efficient (ICC).

Using descriptive statistics, HBPM readings for participants (not taking antihypertensive medication) will be categorised as per European Society of Cardiology guidelines^21^ into:

- Prehypertensive (BP ≥130–134/80–84 mm Hg)
- Hypertensive (BP ≥135–139/85–89 mm Hg)
- Normotensive (BP <130/80 mm Hg)

#### Objective 2: to compare the accuracy of HBPM against ABPM (gold standard) at 12 weeks postpartum

Cohen’s kappa will be used to measure the agreement between HBPM and day ABPM in diagnosing hypertension phenotypes. HBPM will also be compared with night and 24 hours ABPM indices. Accuracy of HBPM will be assessed through sensitivity, specificity, positive and negative predictive values. HBPM will be compared with night and 24 hours ABPM indices to assess. Thresholds for diagnosing hypertension in HBPM will be adjusted to explore the optimal threshold using area under the receiver operating characteristic curve or kappa score. The McNemar’s test will be used to assess whether there is a systematic difference between the HBPM and ABPM for diagnosing hypertension. ICC will be used to check for agreement and Bland-Altman diagrams will also be used to check for random/systematic effects between HBPM and ABPM measurements.

#### Objective 3: to assess the rate of hypertension over the postpartum period (weeks 2–12)

Using descriptive statistics, hypertension rates will be categorised under normotensive (<130/80 mm Hg), prehypertensive (130–135/80–85 mm Hg) or hypertensive (>135/85 mm Hg) at 2-week intervals post birth until 12 weeks postpartum. Univariate and multivariable logistic regression will be used to study what variables are associated with persistent postpartum hypertension at 12 weeks postpartum.

### Questionnaires

The questionnaires will be analysed to answer the remaining objectives:

- The care provided to women at the postnatal GP review in terms of their HDP.
- Participants’ risk perception of CVD.
- Participants’ emotional well-being.
- Participants’ views on HBPM and ABPM.
- Clinicians’ knowledge on HDP and association with CVD development.

Categorical variables will be expressed as frequencies and percentages and continuous variables will be expressed as mean, median, SD and IQR. The data will be analysed and presented using descriptive statistics.

### Ethics and dissemination

This study was approved by London-West London & GTAC Research Ethics Committee. All participants will sign an informed consent form after taking time to read the participant information leaflet. There is a risk of identifying significant hypertension at home and all participants will be counselled on the actions to follow if this were to occur.

The results of this study will be distributed via peer-reviewed academic journals, educational maternity update events for clinicians involved in the care of postpartum women and presenting at conferences and relevant expert meetings. The PPI steering group will be involved in the creation of infographics for a lay audience which will be disseminated among their peer groups and social media platforms to increase the likelihood of key health promotion messages reaching women who have had a hypertensive disorder of pregnancy.

## Supplementary Material

Reviewer comments

Author's manuscript
