# Supplementary material for: Understanding maternal postnatal blood pressure changes following hypertensive disorders in pregnancy: protocol for a prospective cohort study
Source: BMJ Open. 2022 Apr 1;12(4):e060087. doi: 10.1136/bmjopen-2021-060087 (PMC8977789; doi:10.1136/bmjopen-2021-060087)
Supplement: Author's manuscript [file bmjopen-2021-060087.draft_revisions.pdf]

BMJ Open is committed to open peer review. As part of this commitment we make the peer review history of every article we publish publicly available.

When an article is published we post the peer reviewers' comments and the authors' responses online. We also post the versions of the paper that were used during peer review. These are the versions that the peer review comments apply to.

The versions of the paper that follow are the versions that were submitted during the peer review process. They are not the versions of record or the final published versions. They should not be cited or distributed as the published version of this manuscript.

BMJ Open is an open access journal and the full, final, typeset and author-corrected version of record of the manuscript is available on our site with no access controls, subscription charges or pay-per-view fees (http://bmjopen.bmj.com).

If you have any questions on BMJ Open's open peer review process please email info.bmjopen@bmj.com

BMJ Open

Understanding maternal postnatal blood pressure changes following hypertensive disorders in pregnancy: protocol for a prospective cohort study

Journal:	*BMJ Open*
Manuscript ID	bmjopen-2021-060087
Article Type:	Protocol
Date Submitted by the Author:	10-Dec-2021
Complete List of Authors:	Sheehan, Elaine; St George's University of London Molecular and Clinical Sciences Research Institute, Molecular and Clinical Sciences Research Institute; St George's University Hospitals NHS Foundation Trust, Maternal Medicine<br>Wang, Chao; Kingston University Faculty of Health Social Care and Education<br>Cauldwell, Matthew; St George's University Hospitals NHS Foundation Trust, Maternal Medicine<br>Bick, Debra; University of Warwick, Warwick Clinical Trials Unit, Warwick Medical School<br>Thilaganathan, B.; St George's University of London Molecular and Clinical Sciences Research Institute, Fetal Medicine
Keywords:	Hypertension < CARDIOLOGY, Maternal medicine < OBSTETRICS, OBSTETRICS

SCHOLARONE™
Manuscripts

TITLE:

Understanding maternal postnatal blood pressure changes following hypertensive disorders in pregnancy: protocol for a prospective cohort study

Corresponding Author:

Elaine Sheehan, Molecular and Clinical Sciences Research Institute, St George's University of London, London, UK; Maternal Medicine Office, 4th Floor Lanesborough Wing, St. George's University NHS Hospitals, London, SW17 0QT, UK. Email: elaine.sheehan@stgeorges.nhs.uk

Phone Number: 07535558776

Authors:

Chao Wang, Faculty of Health, Social Care and Education, Kingston University London, London, UK.

Matthew Cauldwell, St. George's University NHS Hospitals, London, UK.

Debra Bick, Warwick Clinical Trials Unit, University of Warwick, Warwick, UK.

Basky Thilaganathan, Molecular and Clinical Sciences Research Institute, St George's University of London, London, United Kingdom; Fetal Medicine Unit, Department of Obstetrics and Gynaecology, St George's University Hospitals NHS Foundation Trust, London, UK.

Word Count: 2319

For peer review only

Understanding maternal postnatal blood pressure changes following hypertensive disorders in pregnancy: protocol for a prospective cohort study

Elaine Sheehan, Chao Wang, Debra Bick, Matthew Cauldwell, and Basky Thilaganathan

ABSTRACT

Introduction Hypertensive disorders occur in approximately 10% of women during pregnancy. There is robust population-based data to show that women who have hypertension in pregnancy are much more likely to develop cardiovascular disease (CVD) in the postpartum period. Women with a hypertensive disorder of pregnancy (HDP) are twice more at risk of heart disease and stroke, and four times more likely to develop hypertension after birth. Two out of three women who had HDP will die from CVD. Recent evidence suggests that young women with HDP develop signs of CVD in the immediate postpartum period, rather than several decades later as previously presumed. If confirmed, this concerning finding presents health care practitioners with an opportunity to influence women's cardiovascular health by advising on lifestyle choices and considering therapeutic interventions to prevent the development of CVD.

Methods and Analysis This prospective cohort study design will ask participants to complete three days of home blood pressure monitoring every fortnight for twelve weeks postpartum and will culminate with a 24-hour episode of ambulatory blood pressure monitoring at twelve weeks postpartum. Women and healthcare professionals will complete questionnaires surrounding postpartum care for women who had HDP and knowledge of CVD risk. In addition, the relationship between hypertension and factors likely to influence outcomes such as severity of HDP, maternal age, BMI and ethnicity will be analysed using logistic regression. Blood pressure and data from questionnaires will be analysed using descriptive statistics, with temporal stratification.

Ethics and Dissemination Research ethics approval was obtained from London- West London & GTAC Research Ethics Committee. Research outputs will be published and disseminated through midwifery, obstetric or GP targeted academic journals. The Patient and Public Involvement group will disseminate findings to women who have experienced HDP amongst their peer groups.

For peer review only - http://bmjopen.bmj.com/site/about/guidelines.xhtml

Keywords Hypertensive disorders of pregnancy, postpartum care, cardiovascular disease, pregnancy, blood pressure

ClinicalTrials.gov ID: NCT05137808

ARTICLE SUMMARY

Strengths and Limitations of this study

- This is the first study to our knowledge exploring the recovery of blood pressure longitudinally in the immediate postpartum period after gestational hypertension.
- Reporting of blood pressure at two weekly intervals will inform clinicians of key time points for when blood pressure should be monitored postpartum.
- Self-monitoring and reporting of blood pressure measurements by research participants may introduce the challenge of missing data.

For peer review only

INTRODUCTION

Hypertensive disorders of pregnancy (HDP) affect approximately 10% of pregnant women.[1] The most serious of these disorders is pre-eclampsia which affects 3-5% of pregnant women worldwide. Aside from chronic or pre-existing hypertension which predates pregnancy, gestational hypertension and preeclampsia can occur at any point in pregnancy and early postpartum period from twenty weeks gestation.[1] Women who experience HDP are at increased risk of cardiovascular disease (CVD), hypertension, diabetes, renal disease and stroke in their future life.[1,2] Hypertension is the leading risk factor for CVD in the UK[3] and it can be identified as early as the immediate postpartum period.[4-6] Recent evidence suggests that young women are developing signs of CVD immediately in the postpartum period after HDP, rather than several decades later than previously presumed.[6,7]

Cardiovascular disease is largely preventable with simple lifestyle modifications and treatment.[8] If postpartum women with a history of HDP engaged in healthy lifestyle activities (moderate exercise, reduced alcohol consumption, stopped smoking, healthier diets, reduce time watching television and maintained a healthy body mass index <25), they could reduce their risk of CVD by up to 92%.[9]

There is currently no standard monitoring for CVD development postnatally for women who had HDP, unlike their counterparts - women who develop gestational diabetes (GDM). Women with

GDM are at increased risk of type 2 diabetes in later life. In the postpartum period, GPs assess fasting blood glucose and/or HbA1c to identify the development of type 2 diabetes for women who had GDM and they will receive this annually.[10] Women who have HDP currently do not receive this level of monitoring for the development of CVD. This study seeks to address this disparity by assessing the prevalence of postnatal hypertension which may highlight the need for monitoring of CVD development.

The gold standard for diagnosing hypertension is twenty-four hour ambulatory blood pressure monitoring (ABPM).[11] However, home blood pressure monitoring (HBPM) is becoming more widely used in maternity care, particularly in the antenatal period since the COVID-19 pandemic[12] and more recently, self-management of BP in the postpartum period has been considered.[13] To add to this expanding evidence-base, this study will assess the accuracy of HBPM versus ABPM and if there is a role for HBPM in the postnatal period for diagnosing hypertension.

This study will highlight the proportion of women with persistent hypertension from week two to twelve postpartum and whether these women share common characteristics such as raised BMI and/or maternal age. This is valuable information for clinicians so they know which women are at higher of risk of uncontrolled hypertension and require closer BP surveillance. The current national guidance for monitoring BP in the postpartum period is minimal and this research will therefore ascertain if the national guidance for monitoring hypertension[14] in the postpartum period is sufficient. It will highlight if women who had HDP need further monitoring of hypertension between week two and week eight postpartum and therefore highlight the important timelines for monitoring BP and the need for therapeutic intervention to control BP.

This study will ascertain if the HCPs involved in the care of women who may at be at increased risk of CVD due to developing HDP are educated about the risks and approaches to monitor for CVD. Roth *et al* undertook a systematic review assessing knowledge gaps of women and HCPs around CVD risk and HDP[15] The authors concluded that there was a low level of knowledge among women and HCPs concerning CVD risk and HDP. If HCPs and women are not educated about the risks, the path to significant heart disease is expedited. The postnatal period presents an optimum time for women to be receptive to change and lifestyle modifications[16], with opportunities for HCPs to influence positive change.

The main objective of this prospective cohort study is to observe the recovery (or lack thereof) of maternal hypertension from week two to week twelve after a pregnancy affected by new

For peer review only - http://bmjopen.bmj.com/site/about/guidelines.xhtml

onset of hypertension and identify the prevalence of persistent postpartum hypertension in this cohort of women. A secondary objective will be to assess the accuracy of HBPM in comparison with ABPM to inform if HBPM can be used as an alternative method for diagnosing hypertension in the postpartum period. Other outcomes of interest will include women's self-reporting of their six-to-eight-week GP postnatal review, their perceived risk of developing CVD associated with HDP, their emotional wellbeing and their views on HBPM and ABPM. These objectives will be assessed via online questionnaires distributed at eight and twelve weeks postpartum respectively. In addition, healthcare professionals involved in the postnatal care of women with HDP will receive an online questionnaire assessing their knowledge on HDP and associated CVD risk.

For peer review only

METHODS AND ANALYSIS

Recruitment and Sampling

Participants will be recruited across three NHS maternity sites in South England. Potential participants will be approached during the antenatal and postnatal period before discharge home if they have been identified as having new onset of hypertension in pregnancy by way of convenience sampling. Recruitment will commence in February 2022 until January 2023 and all data collection will cease in April 2023 allowing twelve weeks of blood pressure monitoring from when final participants were recruited in January 2023.

Sample Size

To obtain an accurate estimate of persistent hypertension rate after birth, a sample size of 263 women with a history of HDP are required. The target sample size is 300 women to compensate for study withdrawal and incomplete blood pressure data. This is based on the anticipated rate of persistent postpartum hypertension of approximately 20% and the corresponding width of 95% confidence interval to be 10% as desired level of precision, with 80% probability of achieving such precision. Considerations were also given to the sample size required to assess agreement between Home Blood Pressure Monitoring (HBPM) and Ambulatory Blood Pressure Monitoring (ABPM).

Inclusion and Exclusion Criteria

Eligible research participants will require a diagnosis of new onset hypertension in pregnancy after twenty weeks gestation and up to seventy-two hours postpartum. Research participants in

whom English is not their primary spoken language must demonstrate evidence of the ability to communicate any abnormal BP readings with their healthcare provider.

Patient and Public Involvement

The PPI steering group includes four women from different ethnic groups including Black Caribbean, Asian and White British and European, with a lived experience of HDP who were all involved in discussions related to research priorities and the development of the proposed research study to address priority questions. The PPI group experienced a range of BP disorders during pregnancy including pre-existing hypertension, pre-eclampsia and gestational/postnatal hypertension. This diverse mix of service user backgrounds and experiences ensured that the research team can gain insight into how women prioritise their physical and mental health and wellbeing after birth, and extent to which this may be influenced by their ethnic and cultural backgrounds.

The PPI group have reviewed the research protocol and all research materials (participant information leaflet, consent forms, questionnaires, posters and blood pressure monitoring instructions, study diary) which were revised to reflect their preferences including how information was structured and ensuring language used is suitable for a lay audience. The PPI group have informed the researcher on all stages of the research project since inception, with financial remuneration for their input and time made in line with NIHR guidance.

DATA COLLECTION

Home Blood Pressure Monitoring

Research participants will undergo HBPM every fortnight from week two post birth until week twelve as per a HBPM protocol based on that used by Alemida *et al.* Women will take three BP readings in the morning, early afternoon and in the evening, a total of nine BP readings per day, for three consecutive days.[17] At week twelve, women will undergo twenty-four-hour ABPM (*see* Figure 1).

HBPM will be undertaken using Microlife Watch BP Home as they have been validated for pregnancy and pre-eclampsia.[18] Participants will record their BP measurements manually in a diary provided by the research team. Participants will receive guidance on what to do if they discover BP outside the normal range. Local research midwives at each study site will transfer

For peer review only

the manual BP measurements into electronic format on return of the BP diaries at twelve weeks postpartum.

Ambulatory Blood Pressure Monitoring

Ambulatory blood pressure monitoring will be undertaken using Mobil-O-Graph as these monitors are available to rent, reducing the overall research costs. The Mobil-O-Graph is endorsed by British and Irish Hypertension Society (BIHS) and has been validated against the European Society of Hypertension (ESH) criteria.[19,20] The study site research midwives will upload the twenty-four hour BP analysis to a password protected excel file.

Questionnaires

- **(i)** Questionnaire one will be distributed online at eight weeks postpartum to assess if participants had a postnatal GP review and self-report on what occurred at this appointment if applicable. Part two of the questionnaire will assess participants' risk perception of CVD development and emotional wellbeing following a pregnancy with HDP.
- **(ii)** Questionnaire two will be distributed at twelve-week postpartum assessing participants' views on their experience of HBPM and ABPM. *See* Figure 2.
- **(iii)** A questionnaire aimed at clinicians involved in the care of postpartum women with a HDP will be distributed to midwives, obstetricians and GPs assessing their knowledge on HDP and associated risk of CVD. In addition, a focus group with GPs will provide insight to the postnatal GP check from a clinician's perspective.

DATA ANALYSIS

Objective 1: To identify which BP will be used to calculate the rate of persistent hypertension at twelve weeks postpartum

The first and second BP readings will be compared with the third reading to assess if they vary by more than 10/5mmHg. The mean or median of all BPs will be used if no significance is found or the third BP reading will be used for interpretation if there is a significant difference (>10/5mmHg) between BPs. The BP taken at three time periods (morning, midday and evening) will also be analysed to assess if findings vary significantly by more than 10/5mmHg. If no significance is found, the mean or median BP readings for the day will be calculated and used for further analysis. If significant difference found, each time period will be compared with

For peer review only

daytime ABPM to see which time point mostly correlates with daytime ABPM. This will be analysed using one sample t-test of the difference and intraclass correlation co-efficient (ICC).

Using descriptive statistics, HBPM readings for participants (not taking anti-hypertensive medication) will be categorized as per European Society of Cardiology (ESC) guidelines[21] into:

- Pre-hypertensive (BP ≥130-134/80-84mmHg)
- Hypertensive (BP ≥135-139/85-89mmHg)
- Normotensive (BP <130/80mmHg)

Objective 2: To compare the accuracy of HBPM against ABPM (Gold Standard) at 12 weeks postpartum

Cohen's Kappa will be used to measure the agreement between HBPM and day ABPM in diagnosing hypertension phenotypes. HBPM will also be compared with night and 24hr ABPM indices. Accuracy of HBPM will be assessed through sensitivity, specificity, positive and negative predictive values. HBPM will be compared with night and 24hr ABPM indices to assess. Thresholds for diagnosing hypertension in HBPM will be adjusted to explore the optimal threshold using area under the receiver operating characteristic (ROC) curve or kappa score. The McNemar's test will be used to assess whether there is a systematic difference between the HBPM and ABPM for diagnosing hypertension. ICC will be used to check for agreement and Bland Altman diagrams will also be used to check for random/systematic effects between HBPM and ABPM measurements.

Objective 3: To assess the rate of hypertension over the postpartum period (week 2-12)

Using descriptive statistics, hypertension rates will be categorized under normotensive (<130/80mmHg), pre-hypertensive (130-135/80-85mmHg) or hypertensive (>135/85) at two-week intervals post birth until twelve weeks postpartum. Univariate and multivariable logistic regression will be used to study what variables are associated with persistent postpartum hypertension at twelve weeks postpartum.

Questionnaires

The questionnaires will be analysed to answer the remaining objectives:

- the care provided to women at the postnatal GP review in terms of their HDP
- participants' risk perception of CVD

For peer review only

- participants' emotional wellbeing
- participants' views on HBPM and ABPM
- clinicians' knowledge on HDP and association with CVD development

Categorical variables will be expressed as frequencies and percentages and continuous variables will be expressed as mean, median, standard deviations and interquartile range. The data will be analysed and presented using descriptive statistics.

ETHICS

This study was approved by London- West London & GTAC Research Ethics Committee. All participants will sign an informed consent form after taking time to read the participant information leaflet. There is a risk of identifying significant hypertension at home and all participants will be counselled on the actions to follow if this were to occur.

DISSEMINATION

The results of this study will be distributed via peer reviewed academic journals, educational maternity update events for clinicians involved in the care of postpartum women and presenting at conferences and relevant expert meetings. The PPI steering group will be involved in the creation of infographics for a lay audience which will be disseminated amongst their peer groups and social media platforms to increase the likelihood of key health promotion messages reaching women who have had a hypertensive disorder of pregnancy.

ACKNOWLEDGEMENTS

The research team would like to acknowledge the effort and input from the PPI steering group which was integral to the development of this proposed research.

AUTHOR STATEMENT

ES is the leading author and doctoral student of this study. CW is a senior statistician and has supervised the development of the data analysis plan. DB is Professor of Maternal Health and has advised on the questionnaire content and development. MC and BT are consultant obstetricians and have advised on the data collection and analysis of BP monitoring. CW, DB, MC and BT form the supervisory team for the doctoral student and all have contributed to the study design. All authors have reviewed and agreed the final protocol.

For peer review only

FUNDING STATEMENT

This work is funded by National Institute of Health Research (NIHR), grant number NIHR301587.

CONFLICTS OF INTERESTS STATEMENT

The authors have no competing interest to declare.

For peer review only

REFERENCES

1. Benschop L, Duvekot J and Routers van Lennep J. Future risk of cardiovascular disease risk factors and events in women after a hypertensive disorder of pregnancy, *Heart*, 2019; 105:1273–1278. doi:10.1136/heartjnl-2018-313453
2. Preeclampsia Foundation. 'Preeclampsia and future cardiovascular disease in women: what do we know and what can we do?', Preeclampsia Foundation Position Paper, 2019.
3. British Heart Foundation. 'Heart Statistics: UK Fact Sheet', 2020.
4. Goel A, Maski M, Bajracharya S, *et al*., 'Epidemiology and mechanisms of de novo and persistent hypertension in the postpartum period', *Circulation,* 2015; 142:1726-1733. Doi: https://10.1161/circulayionaha.115.015721
5. Ditisheim A, Wuerzner G, Ponte B, Vial Y, Irion O, Burnier M, *et al*. 'Prevalence of hypertneisve phenotypes after preeclampsia: a prospective cohort study', *Hypertension,* 2017; 71: 103-109. Doi: https://10.1161/hypertensionaha.117.09799
6. Giorgione V, Ridder, A, Kalaflat, E, *et al*. Incidence of postpartum hypertension within 2 years of a pregnancy complicated by pre-eclampsia: a systematic review and meta-analysis, *British Journal of Obstetrics and Gynaecology*, 2021; 128 (3): 495-503.
7. Cain MA, Salemi JL, Tanner JP, *et al*. 'Pregnancy as a window to future health: maternal placental syndromes and short-term cardiovascular outcomes', *American Journal of Obstetrics and Gynaecology,* 2016; 215 (4). Doi: https://10.1016/j.ajog.2016.05.047
8. Eckel RH, Jakicic, JM, Ard, JD, *et al*, '2013 AHA/ACC guideline on lifestyle management to reduce cardiovascular risk', *Circulation,* 2014;129: 76-99. Doi: 10.1161/01.cir.0000437740.48606.d1.
9. Timpka S, Stuart J, Tanz LJ, *et al*. 'Lifestyle in progression from hypertensive disorders of pregnancy to chronic hypertension in nurses' health study II: observational cohort study', *British Medical Journal*, 2017; 358; j3024. doi: https://doi.org/10.1136/bmj.j3024
10. NICE. Diabetes in pregnancy: management from preconception to postnatal period, 2015; NG3.
11. O' Brien E, Parati G, Stergiou G, *et al.* European Society of Hypertension position paper on ambulatory blood pressure monitoring. *Journal of Hypertension,* 2013; 31(9): 1731-1768. Doi:10.1097/HJH.0b013e328363e964
12. Fletcher B, Chappell L, Lavallee L, *et al*. Changes to management of hypertension in pregnancy, and attitudes to self-management: An online survey of obstetricians, before and following the first wave of the COVID-19 pandemic, *Pregnancy Hypertension*, 2021; 26: 54-61. https://doi.org/10.1016/j.preghy.2021.08.117

13. Cairns AE, Tucker KL, Leeson P, *et al*. Self-Management of Postnatal Hypertension: The SNAP-HT Trial, *Hypertension.* 2018;72:425–432. https://doi.org/10.1161/HYPERTENSIONAHA.118.10911
14. NICE. Hypertension in pregnancy CKS: Postpartum follow-up for hypertensive disorders in pregnancy, 2019.
15. Roth H, LeMarquand G, Henry A *et al*. Assessing knowledge gaps of women and healthcare providers concerning cardiovascular risk after hypertensive disorders of pregnancy- a systematic review, *Frontiers in Cardiovascular Medicine.* 2019*;* 6 (178). Doi: https://doi.org/10.3389/fcvm.2019.00178
16. Phelan S. Pregnancy: a 'teachable moment' for weight control and obesity prevention. *American Journal of Obstetrics and Gynaecology.* 2010; 202 (2).
17. Almeida A, Stein R, Gus M, *et al*. Improved diagnostic accuracy of a 3-day protocol of home blood pressure monitoring for the diagnosis of arterial hypertension. *Blood Pressure Monitoring,* 2013; 18:119-126. Doi: https://10.1097/mbp.0b013e32835ebb18
18. Clark K, Snowball O, Nzelu D, *et al*. Validation of the microlife watchBP home blood pressure device in pregnancy for medium and large arm circumferences, *Blood Pressure Monitoring,* 2018*;* 23 (3): 171-174. Doi: https://10.1097/mbp.0000000000000315
19. Franssen PML and Imholz BPM. Evaluation of the Mobil-O-Graph new generation ABPM device using the ESH criteria. *Blood Pressure Monitoring*. 2010; 15: 229-231
20. Weiss W, Tolle M, Zidek, W and van der Giet M. Validation of the Mobil-O-Graph: 24h-blood pressure measurement device. *Blood Pressure Monitoring*. 2010; 15: 225-228.
21. Williams B, Mancia G, Spiering W, *et al*. ESC/ESH Guidelines for the management of arterial hypertension. *European Heart Journal*. 2018; 39: 3021-3104.

For peer review only

Figure 1: Overview of participant blood pressure monitoring protocol

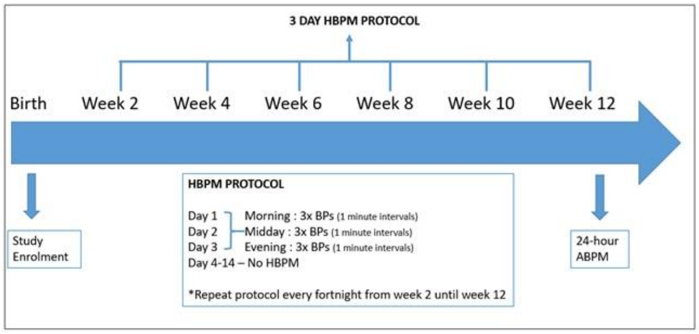

review only

Figure 2: Timeline of events for research participant

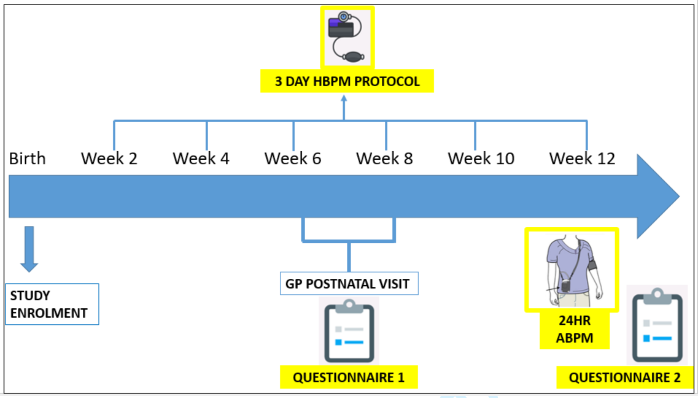

For peer review only

BMJ Open

Understanding maternal postnatal blood pressure changes following hypertensive disorders in pregnancy: protocol for a prospective cohort study

Journal:	*BMJ Open*
Manuscript ID	bmjopen-2021-060087.R1
Article Type:	Protocol
Date Submitted by the Author:	07-Feb-2022
Complete List of Authors:	Sheehan, Elaine; St George's University of London Molecular and Clinical Sciences Research Institute, Molecular and Clinical Sciences Research Institute; St George's University Hospitals NHS Foundation Trust, Maternal Medicine<br>Wang, Chao; Kingston University Faculty of Health Social Care and Education<br>Cauldwell, Matthew; St George's University Hospitals NHS Foundation Trust, Maternal Medicine<br>Bick, Debra; University of Warwick, Warwick Clinical Trials Unit, Warwick Medical School<br>Thilaganathan, B.; St George's University of London Molecular and Clinical Sciences Research Institute, Fetal Medicine
**Primary Subject Heading**:	Obstetrics and gynaecology
Secondary Subject Heading:	Cardiovascular medicine
Keywords:	Hypertension < CARDIOLOGY, Maternal medicine < OBSTETRICS, OBSTETRICS

SCHOLARONE™
Manuscripts

TITLE:

Understanding maternal postnatal blood pressure changes following hypertensive disorders in pregnancy: protocol for a prospective cohort study

Corresponding Author:

Elaine Sheehan, Molecular and Clinical Sciences Research Institute, St George's University of London, London, UK; Maternal Medicine Office, 4th Floor Lanesborough Wing, St. George's University NHS Hospitals, London, SW17 0QT, UK. Email: elaine.sheehan@stgeorges.nhs.uk

Phone Number: 07535558776

Authors:

Chao Wang, Faculty of Health, Social Care and Education, Kingston University London, London, UK.

Matthew Cauldwell, St. George's University NHS Hospitals, London, UK.

Debra Bick, Warwick Clinical Trials Unit, University of Warwick, Warwick, UK.

Basky Thilaganathan, Molecular and Clinical Sciences Research Institute, St George's University of London, London, United Kingdom; Fetal Medicine Unit, Department of Obstetrics and Gynaecology, St George's University Hospitals NHS Foundation Trust, London, UK.

Word Count: 2320

For peer review only

Understanding maternal postnatal blood pressure changes following hypertensive disorders in pregnancy: protocol for a prospective cohort study

Elaine Sheehan, Chao Wang, Debra Bick, Matthew Cauldwell, and Basky Thilaganathan

ABSTRACT

Introduction Hypertensive disorders occur in approximately 10% of women during pregnancy. There is robust population-based data to show that women who have hypertension in pregnancy are much more likely to develop cardiovascular disease (CVD) in the postpartum period. Women with a hypertensive disorder of pregnancy (HDP) are twice more at risk of heart disease and stroke, and four times more likely to develop hypertension after birth. Two out of three women who had HDP will die from CVD. Recent evidence suggests that young women with HDP develop signs of CVD in the immediate postpartum period, rather than several decades later as previously presumed. If confirmed, this concerning finding presents health care practitioners with an opportunity to influence women's cardiovascular health by advising on lifestyle choices and considering therapeutic interventions to prevent the development of CVD.

Methods and Analysis This prospective cohort study design will ask approximately 300 participants to complete three days of home blood pressure monitoring every fortnight for twelve weeks postpartum and will culminate with a 24-hour episode of ambulatory blood pressure monitoring at twelve weeks postpartum. Women and healthcare professionals will complete questionnaires surrounding postpartum care for women who had HDP and knowledge of CVD risk. In addition, the relationship between hypertension and factors likely to influence outcomes such as severity of HDP, maternal age, BMI and ethnicity will be analysed using logistic regression. Blood pressure and data from questionnaires will be analysed using descriptive statistics, with temporal stratification.

Ethics and Dissemination Research ethics approval was obtained from London- West London & GTAC Research Ethics Committee. Research outputs will be published and disseminated through midwifery, obstetric or GP targeted academic journals. The Patient and Public Involvement group will disseminate findings to women who have experienced HDP amongst their peer groups.

For peer review only

Keywords Hypertensive disorders of pregnancy, postpartum care, cardiovascular disease, pregnancy, blood pressure

ClinicalTrials.gov ID: NCT05137808

ARTICLE SUMMARY

Strengths and Limitations of this study

- This is the first study to our knowledge exploring the recovery of blood pressure longitudinally in the immediate postpartum period after gestational hypertension.
- Home blood pressure monitoring will be compared against the gold standard; ambulatory blood pressure monitoring
- Self-monitoring and reporting of blood pressure measurements by research participants may introduce the challenge of missing data.

INTRODUCTION

Hypertensive disorders of pregnancy (HDP) affect approximately 10% of pregnant women.[1] The most serious of these disorders is pre-eclampsia which affects 3-5% of pregnant women worldwide. Aside from chronic or pre-existing hypertension which predates pregnancy, gestational hypertension and preeclampsia can occur at any point in pregnancy and early postpartum period from twenty weeks gestation.[1] Women who experience HDP are at increased risk of cardiovascular disease (CVD), hypertension, diabetes, renal disease and stroke in their future life.[1,2] Hypertension is the leading risk factor for CVD in the UK[3] and it can be identified as early as the immediate postpartum period.[4-6] Recent evidence suggests that young women are developing signs of CVD immediately in the postpartum period after HDP, rather than several decades later than previously presumed.[6,7]

Cardiovascular disease is largely preventable with simple lifestyle modifications and treatment.[8] If postpartum women with a history of HDP engaged in healthy lifestyle activities (moderate exercise, reduced alcohol consumption, stopped smoking, healthier diets, reduce time watching television and maintained a healthy body mass index <25), they could reduce their risk of CVD by up to 92%.[9]

There is currently no standard monitoring for CVD development postnatally for women who had HDP, unlike their counterparts - women who develop gestational diabetes (GDM). Women with

For peer review only

GDM are at increased risk of type 2 diabetes in later life. In the postpartum period, GPs assess fasting blood glucose and/or HbA1c to identify the development of type 2 diabetes for women who had GDM and they will receive this annually.[10] Women who have HDP currently do not receive this level of monitoring for the development of CVD. This study seeks to address this disparity by assessing the prevalence of postnatal hypertension which may highlight the need for monitoring of CVD development.

The gold standard for diagnosing hypertension is twenty-four hour ambulatory blood pressure monitoring (ABPM).[11] However, home blood pressure monitoring (HBPM) is becoming more widely used in maternity care, particularly in the antenatal period since the COVID-19 pandemic[12] and more recently, self-management of BP in the postpartum period has been considered.[13] To add to this expanding evidence-base, this study will assess the accuracy of HBPM versus ABPM and if there is a role for HBPM in the postnatal period for diagnosing hypertension.

This study will highlight the proportion of women with persistent hypertension from week two to twelve postpartum and whether these women share common characteristics such as raised BMI and/or maternal age. This is valuable information for clinicians so they know which women are at higher of risk of uncontrolled hypertension and require closer BP surveillance. The current national guidance for monitoring BP in the postpartum period is minimal and this research will therefore ascertain if the national guidance for monitoring hypertension[14] in the postpartum period is sufficient. It will highlight if women who had HDP need further monitoring of hypertension between week two and week eight postpartum and therefore highlight the important timelines for monitoring BP and the need for therapeutic intervention to control BP.

This study will ascertain if the HCPs involved in the care of women who may at be at increased risk of CVD due to developing HDP are educated about the risks and approaches to monitor for CVD. Roth *et al* undertook a systematic review assessing knowledge gaps of women and HCPs around CVD risk and HDP[15] The authors concluded that there was a low level of knowledge among women and HCPs concerning CVD risk and HDP. If HCPs and women are not educated about the risks, the path to significant heart disease is expedited. The postnatal period presents an optimum time for women to be receptive to change and lifestyle modifications[16], with opportunities for HCPs to influence positive change.

The main objective of this prospective cohort study is to observe the recovery (or lack thereof) of maternal hypertension from week two to week twelve after a pregnancy affected by new

For peer review only

onset of hypertension and identify the prevalence of persistent postpartum hypertension in this cohort of women. A secondary objective will be to assess the accuracy of HBPM in comparison with ABPM to inform if HBPM can be used as an alternative method for diagnosing hypertension in the postpartum period. Other outcomes of interest will include women's self-reporting of their six-to-eight-week GP postnatal review, their perceived risk of developing CVD associated with HDP, their emotional wellbeing and their views on HBPM and ABPM. These objectives will be assessed via online questionnaires distributed at eight and twelve weeks postpartum respectively. In addition, healthcare professionals involved in the postnatal care of women with HDP will receive an online questionnaire assessing their knowledge on HDP and associated CVD risk.

METHODS AND ANALYSIS

Recruitment and Sampling

Participants will be recruited across three NHS maternity sites in South England. Potential participants will be approached during the antenatal and postnatal period before discharge home if they have been identified as having new onset of hypertension in pregnancy by way of convenience sampling. Recruitment will commence in April2022 until March 2023 and all data collection will cease in June 2023 allowing twelve weeks of blood pressure monitoring from when final participants were recruited in March 2023.

Sample Size

To obtain an accurate estimate of persistent hypertension rate after birth, a sample size of 263 women with a history of HDP are required. The target sample size is 300 women to compensate for study withdrawal and incomplete blood pressure data. This is based on the anticipated rate of persistent postpartum hypertension of approximately 20% and the corresponding width of 95% confidence interval to be 10% as desired level of precision, with 80% probability of achieving such precision. Considerations were also given to the sample size required to assess agreement between Home Blood Pressure Monitoring (HBPM) and Ambulatory Blood Pressure Monitoring (ABPM).

Inclusion and Exclusion Criteria

Eligible research participants will require a diagnosis of new onset hypertension in pregnancy after twenty weeks gestation and up to seventy-two hours postpartum. Research participants in

For peer review only

whom English is not their primary spoken language must demonstrate evidence of the ability to communicate any abnormal BP readings with their healthcare provider.

Patient and Public Involvement

The PPI steering group includes four women from different ethnic groups including Black Caribbean, Asian and White British and European, with a lived experience of HDP who were all involved in discussions related to research priorities and the development of the proposed research study to address priority questions. The PPI group experienced a range of BP disorders during pregnancy including pre-existing hypertension, pre-eclampsia and gestational/postnatal hypertension. This diverse mix of service user backgrounds and experiences ensured that the research team can gain insight into how women prioritise their physical and mental health and wellbeing after birth, and extent to which this may be influenced by their ethnic and cultural backgrounds.

The PPI group have reviewed the research protocol and all research materials (participant information leaflet, consent forms, questionnaires, posters and blood pressure monitoring instructions, study diary) which were revised to reflect their preferences including how information was structured and ensuring language used is suitable for a lay audience. The PPI group have informed the researcher on all stages of the research project since inception, with financial remuneration for their input and time made in line with NIHR guidance.

DATA COLLECTION

Home Blood Pressure Monitoring

Research participants will undergo HBPM every fortnight from week two post birth until week twelve as per a HBPM protocol based on that used by Alemida *et al.* Women will take three BP readings in the morning, early afternoon and in the evening, a total of nine BP readings per day, for three consecutive days.[17] At week twelve, women will undergo twenty-four-hour ABPM (*see* Figure 1).

HBPM will be undertaken using Microlife Watch BP Home as they have been validated for pregnancy and pre-eclampsia.[18] Participants will record their BP measurements manually in a diary provided by the research team. Participants will receive guidance on what to do if they discover BP outside the normal range. Local research midwives at each study site will transfer

For peer review only

the manual BP measurements into electronic format on return of the BP diaries at twelve weeks postpartum.

Ambulatory Blood Pressure Monitoring

Ambulatory blood pressure monitoring will be undertaken using Mobil-O-Graph as these monitors are available to rent, reducing the overall research costs. The Mobil-O-Graph is endorsed by British and Irish Hypertension Society (BIHS) and has been validated against the European Society of Hypertension (ESH) criteria.[19,20] The study site research midwives will upload the twenty-four hour BP analysis to a password protected excel file.

Questionnaires

(i) Questionnaire one will be distributed online at eight weeks postpartum to assess if participants had a postnatal GP review and self-report on what occurred at this appointment if applicable. Part two of the questionnaire will assess participants' risk perception of CVD development and emotional wellbeing following a pregnancy with HDP.

(ii) Questionnaire two will be distributed at twelve-week postpartum assessing participants' views on their experience of HBPM and ABPM. *See* Figure 2.

(iii) A questionnaire aimed at clinicians involved in the care of postpartum women with a HDP will be distributed to midwives, obstetricians and GPs assessing their knowledge on HDP and associated risk of CVD. In addition, a focus group with GPs will provide insight to the postnatal GP check from a clinician's perspective.

DATA ANALYSIS

Objective 1: To identify which BP will be used to calculate the rate of persistent hypertension at twelve weeks postpartum

The first and second BP readings will be compared with the third reading to assess if they vary by more than 10/5mmHg. The mean or median of all BPs will be used if no significance is found or the third BP reading will be used for interpretation if there is a significant difference (>10/5mmHg) between BPs. The BP taken at three time periods (morning, midday and evening) will also be analysed to assess if findings vary significantly by more than 10/5mmHg. If no significance is found, the mean or median BP readings for the day will be calculated and used for further analysis. If significant difference found, each time period will be compared with

For peer review only

daytime ABPM to see which time point mostly correlates with daytime ABPM. This will be analysed using one sample t-test of the difference and intraclass correlation co-efficient (ICC).

Using descriptive statistics, HBPM readings for participants (not taking anti-hypertensive medication) will be categorized as per European Society of Cardiology (ESC) guidelines[21] into:

- Pre-hypertensive (BP ≥130-134/80-84mmHg)
- Hypertensive (BP ≥135-139/85-89mmHg)
- Normotensive (BP <130/80mmHg)

Objective 2: To compare the accuracy of HBPM against ABPM (Gold Standard) at 12 weeks postpartum

Cohen's Kappa will be used to measure the agreement between HBPM and day ABPM in diagnosing hypertension phenotypes. HBPM will also be compared with night and 24hr ABPM indices. Accuracy of HBPM will be assessed through sensitivity, specificity, positive and negative predictive values. HBPM will be compared with night and 24hr ABPM indices to assess. Thresholds for diagnosing hypertension in HBPM will be adjusted to explore the optimal threshold using area under the receiver operating characteristic (ROC) curve or kappa score. The McNemar's test will be used to assess whether there is a systematic difference between the HBPM and ABPM for diagnosing hypertension. ICC will be used to check for agreement and Bland Altman diagrams will also be used to check for random/systematic effects between HBPM and ABPM measurements.

Objective 3: To assess the rate of hypertension over the postpartum period (week 2-12)

Using descriptive statistics, hypertension rates will be categorized under normotensive (<130/80mmHg), pre-hypertensive (130-135/80-85mmHg) or hypertensive (>135/85) at two-week intervals post birth until twelve weeks postpartum. Univariate and multivariable logistic regression will be used to study what variables are associated with persistent postpartum hypertension at twelve weeks postpartum.

Questionnaires

The questionnaires will be analysed to answer the remaining objectives:

- the care provided to women at the postnatal GP review in terms of their HDP
- participants' risk perception of CVD

For peer review only

- participants' emotional wellbeing
- participants' views on HBPM and ABPM
- clinicians' knowledge on HDP and association with CVD development

Categorical variables will be expressed as frequencies and percentages and continuous variables will be expressed as mean, median, standard deviations and interquartile range. The data will be analysed and presented using descriptive statistics.

ETHICS AND DISSEMINATION

This study was approved by London- West London & GTAC Research Ethics Committee. All participants will sign an informed consent form after taking time to read the participant information leaflet. There is a risk of identifying significant hypertension at home and all participants will be counselled on the actions to follow if this were to occur.

The results of this study will be distributed via peer reviewed academic journals, educational maternity update events for clinicians involved in the care of postpartum women and presenting at conferences and relevant expert meetings. The PPI steering group will be involved in the creation of infographics for a lay audience which will be disseminated amongst their peer groups and social media platforms to increase the likelihood of key health promotion messages reaching women who have had a hypertensive disorder of pregnancy.

ACKNOWLEDGEMENTS

The research team would like to acknowledge the effort and input from the PPI steering group which was integral to the development of this proposed research.

AUTHOR STATEMENT

ES is the leading author and doctoral student of this study. CW is a senior statistician and has supervised the development of the data analysis plan. DB is Professor of Maternal Health and has advised on the questionnaire content and development. MC and BT are consultant obstetricians and have advised on the data collection and analysis of BP monitoring. CW, DB, MC and BT form the supervisory team for the doctoral student and all have contributed to the study design. All authors have reviewed and agreed the final protocol.

FUNDING STATEMENT

For peer review only

This work is funded by National Institute of Health Research (NIHR), grant number NIHR301587.

CONFLICTS OF INTERESTS STATEMENT

The authors have no competing interest to declare.

For peer review only

REFERENCES

1. Benschop L, Duvekot J and Routers van Lennep J. Future risk of cardiovascular disease risk factors and events in women after a hypertensive disorder of pregnancy, *Heart*, 2019; 105:1273–1278. doi:10.1136/heartjnl-2018-313453
2. Preeclampsia Foundation. 'Preeclampsia and future cardiovascular disease in women: what do we know and what can we do?', Preeclampsia Foundation Position Paper, 2019.
3. British Heart Foundation. 'Heart Statistics: UK Fact Sheet', 2020.
4. Goel A, Maski M, Bajracharya S, *et al*., 'Epidemiology and mechanisms of de novo and persistent hypertension in the postpartum period', *Circulation,* 2015; 142:1726-1733. Doi: https://10.1161/circulayionaha.115.015721
5. Ditisheim A, Wuerzner G, Ponte B, Vial Y, Irion O, Burnier M, *et al*. 'Prevalence of hypertneisve phenotypes after preeclampsia: a prospective cohort study', *Hypertension,* 2017; 71: 103-109. Doi: https://10.1161/hypertensionaha.117.09799
6. Giorgione V, Ridder, A, Kalaflat, E, *et al*. Incidence of postpartum hypertension within 2 years of a pregnancy complicated by pre-eclampsia: a systematic review and meta-analysis, *British Journal of Obstetrics and Gynaecology*, 2021; 128 (3): 495-503.
7. Cain MA, Salemi JL, Tanner JP, *et al*. 'Pregnancy as a window to future health: maternal placental syndromes and short-term cardiovascular outcomes', *American Journal of Obstetrics and Gynaecology,* 2016; 215 (4). Doi: https://10.1016/j.ajog.2016.05.047
8. Eckel RH, Jakicic, JM, Ard, JD, *et al*, '2013 AHA/ACC guideline on lifestyle management to reduce cardiovascular risk', *Circulation,* 2014;129: 76-99. Doi: 10.1161/01.cir.0000437740.48606.d1.
9. Timpka S, Stuart J, Tanz LJ, *et al*. 'Lifestyle in progression from hypertensive disorders of pregnancy to chronic hypertension in nurses' health study II: observational cohort study', *British Medical Journal*, 2017; 358; j3024. doi: https://doi.org/10.1136/bmj.j3024
10. NICE. Diabetes in pregnancy: management from preconception to postnatal period, 2015; NG3.
11. O' Brien E, Parati G, Stergiou G, *et al.* European Society of Hypertension position paper on ambulatory blood pressure monitoring. *Journal of Hypertension,* 2013; 31(9): 1731-1768. Doi:10.1097/HJH.0b013e328363e964
12. Fletcher B, Chappell L, Lavallee L, *et al*. Changes to management of hypertension in pregnancy, and attitudes to self-management: An online survey of obstetricians, before and following the first wave of the COVID-19 pandemic, *Pregnancy Hypertension*, 2021; 26: 54-61. https://doi.org/10.1016/j.preghy.2021.08.117

13. Cairns AE, Tucker KL, Leeson P, *et al*. Self-Management of Postnatal Hypertension: The SNAP-HT Trial, *Hypertension.* 2018;72:425–432. https://doi.org/10.1161/HYPERTENSIONAHA.118.10911
14. NICE. Hypertension in pregnancy CKS: Postpartum follow-up for hypertensive disorders in pregnancy, 2019.
15. Roth H, LeMarquand G, Henry A *et al*. Assessing knowledge gaps of women and healthcare providers concerning cardiovascular risk after hypertensive disorders of pregnancy- a systematic review, *Frontiers in Cardiovascular Medicine.* 2019*;* 6 (178). Doi: https://doi.org/10.3389/fcvm.2019.00178
16. Phelan S. Pregnancy: a 'teachable moment' for weight control and obesity prevention. *American Journal of Obstetrics and Gynaecology.* 2010; 202 (2).
17. Almeida A, Stein R, Gus M, *et al*. Improved diagnostic accuracy of a 3-day protocol of home blood pressure monitoring for the diagnosis of arterial hypertension. *Blood Pressure Monitoring,* 2013; 18:119-126. Doi: https://10.1097/mbp.0b013e32835ebb18
18. Clark K, Snowball O, Nzelu D, *et al*. Validation of the microlife watchBP home blood pressure device in pregnancy for medium and large arm circumferences, *Blood Pressure Monitoring,* 2018*;* 23 (3): 171-174. Doi: https://10.1097/mbp.0000000000000315
19. Franssen PML and Imholz BPM. Evaluation of the Mobil-O-Graph new generation ABPM device using the ESH criteria. *Blood Pressure Monitoring*. 2010; 15: 229-231
20. Weiss W, Tolle M, Zidek, W and van der Giet M. Validation of the Mobil-O-Graph: 24h-blood pressure measurement device. *Blood Pressure Monitoring*. 2010; 15: 225-228.
21. Williams B, Mancia G, Spiering W, *et al*. ESC/ESH Guidelines for the management of arterial hypertension. *European Heart Journal*. 2018; 39: 3021-3104.

For peer review only

Figure 1: Overview of participant blood pressure monitoring protocol

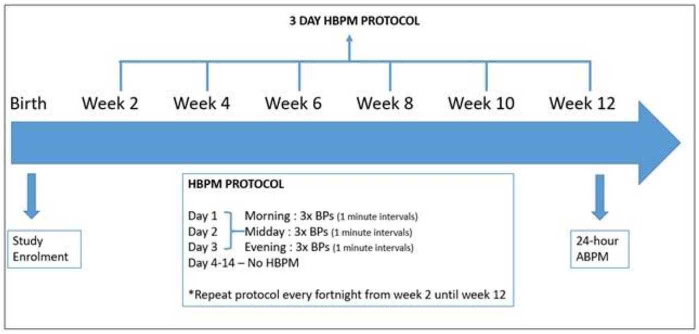

Figure 2: Timeline of events for research participant

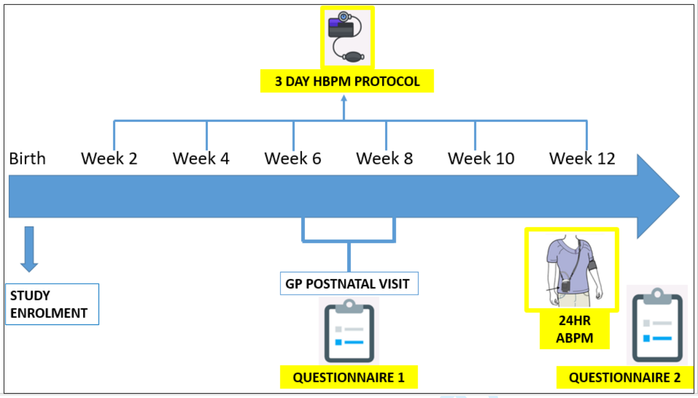

STROBE Statement—checklist of items that should be included in reports of observational studies

	Item No.	Recommendation	Page No.	Relevant text from manuscript
**Title and abstract**	1	(*a*) Indicate the study's design with a commonly used term in the title or the abstract	2	Line 33
		(*b*) Provide in the abstract an informative and balanced summary of what was done and what was found	NA	NA
**Introduction**				
Background/rationale	2	Explain the scientific background and rationale for the investigation being reported	3<br>4	Line 32-55<br>Line 3-26
Objectives	3	State specific objectives, including any prespecified hypotheses	4<br>5	Line 28-55<br>Line 3-21
**Methods**				
Study design	4	Present key elements of study design early in the paper	5	Line 3-8
Setting	5	Describe the setting, locations, and relevant dates, including periods of recruitment, exposure, follow-up, and data collection	5	Line 28-53
Participants	6	(*a*) *Cohort study*—Give the eligibility criteria, and the sources and methods of selection of participants. Describe methods of follow-up<br>*Case-control study*—Give the eligibility criteria, and the sources and methods of case ascertainment and control selection. Give the rationale for the choice of cases and controls<br>*Cross-sectional study*—Give the eligibility criteria, and the sources and methods of selection of participants	6	Line 3-8
		(*b*) *Cohort study*—For matched studies, give matching criteria and number of exposed and unexposed<br>*Case-control study*—For matched studies, give matching criteria and the number of controls per case	NA	NA
Variables	7	Clearly define all outcomes, exposures, predictors, potential confounders, and effect modifiers. Give diagnostic criteria, if applicable	8	Line 16-19
Data sources/ measurement	8*	For each variable of interest, give sources of data and details of methods of assessment (measurement). Describe comparability of assessment methods if there is more than one group	*NA*	*NA*
Bias	9	Describe any efforts to address potential sources of bias	NA	NA

For peer review only

Study size	10	Explain how the study size was arrived at	5	Line 42-53

Continued on next page

For peer review only

Quantitative variables	11	Explain how quantitative variables were handled in the analyses. If applicable, describe which groupings were chosen and why	NA	NA
Statistical methods	12	(*a*) Describe all statistical methods, including those used to control for confounding	NA	NA
		(*b*) Describe any methods used to examine subgroups and interactions	NA	NA
		(*c*) Explain how missing data were addressed	NA	NA
		(*d*) *Cohort study*—If applicable, explain how loss to follow-up was addressed<br>*Case-control study*—If applicable, explain how matching of cases and controls was addressed<br>*Cross-sectional study*—If applicable, describe analytical methods taking account of sampling strategy	NA	NA
		(*e*) Describe any sensitivity analyses	NA	NA
**Results**				
Participants	13*	(a) Report numbers of individuals at each stage of study—eg numbers potentially eligible, examined for eligibility, confirmed eligible, included in the study, completing follow-up, and analysed	NA	NA
		(b) Give reasons for non-participation at each stage	NA	NA
		(c) Consider use of a flow diagram	NA	NA
Descriptive data	14*	(a) Give characteristics of study participants (eg demographic, clinical, social) and information on exposures and potential confounders	NA	NA
		(b) Indicate number of participants with missing data for each variable of interest	NA	NA
		(c) *Cohort study*—Summarise follow-up time (eg, average and total amount)	NA	NA
Outcome data	15*	*Cohort study*—Report numbers of outcome events or summary measures over time	NA	NA
		Case-control study—Report numbers in each exposure category, or summary measures of exposure	NA	NA
		Cross-sectional study—Report numbers of outcome events or summary measures	NA	NA
Main results	16	(*a*) Give unadjusted estimates and, if applicable, confounder-adjusted estimates and their precision (eg, 95% confidence interval). Make clear which confounders were adjusted for and why they were included	NA	NA
		(*b*) Report category boundaries when continuous variables were categorized	NA	NA
		(*c*) If relevant, consider translating estimates of relative risk into absolute risk for a meaningful time period	NA	NA

Continued on next page

For peer review only - http://bmjopen.bmj.com/site/about/guidelines.xhtml

Other analyses	17	Report other analyses done—eg analyses of subgroups and interactions, and sensitivity analyses	NA	NA
**Discussion**				
Key results	18	Summarise key results with reference to study objectives	NA	NA
Limitations	19	Discuss limitations of the study, taking into account sources of potential bias or imprecision. Discuss both direction and magnitude of any potential bias	NA	NA
Interpretation	20	Give a cautious overall interpretation of results considering objectives, limitations, multiplicity of analyses, results from similar studies, and other relevant evidence	NA	NA
Generalisability	21	Discuss the generalisability (external validity) of the study results	NA	NA
**Other information**				
Funding	22	Give the source of funding and the role of the funders for the present study and, if applicable, for the original study on which the present article is based	10	Line 9-13

*Give information separately for cases and controls in case-control studies and, if applicable, for exposed and unexposed groups in cohort and cross-sectional studies.

Note: An Explanation and Elaboration article discusses each checklist item and gives methodological background and published examples of transparent reporting. The STROBE checklist is best used in conjunction with this article (freely available on the Web sites of PLoS Medicine at http://www.plosmedicine.org/, Annals of Internal Medicine at http://www.annals.org/, and Epidemiology at http://www.epidem.com/). Information on the STROBE Initiative is available at www.strobe-statement.org.

For peer review only
