# Supplementary material for: Understanding maternal postnatal blood pressure changes following hypertensive disorders in pregnancy: protocol for a prospective cohort study
Source: BMJ Open. 2022 Apr 1;12(4):e060087. doi: 10.1136/bmjopen-2021-060087 (PMC8977789; doi:10.1136/bmjopen-2021-060087)
Supplement: Reviewer comments [file bmjopen-2021-060087.reviewer_comments.pdf]

PEER REVIEW HISTORY

BMJ Open publishes all reviews undertaken for accepted manuscripts. Reviewers are asked to complete a checklist review form (**http://bmjopen.bmj.com/site/about/resources/checklist.pdf**) and are provided with free text boxes to elaborate on their assessment. These free text comments are reproduced below.

ARTICLE DETAILS

**TITLE (PROVISIONAL)**	Understanding maternal postnatal blood pressure changes following hypertensive disorders in pregnancy: protocol for a prospective cohort study
**AUTHORS**	Sheehan, Elaine; Wang, Chao; Cauldwell, Matthew; Bick, Debra; Thilaganathan, B.

This article was not externally reviewed at BMJ Open. Protocols that have been independently assessed prior to submission to BMJ Open are usually fast-tracked to publication on the grounds that further substantial changes will not be possible. This independent assessment will usually be external, independent review for both a substantial grant award from a non-commercial or government funder and ethics approval.
